# Effects of Dietary DL-Methionine Supplementation on Meat Quality and Antioxidant Capacity in Wenchang Chickens Subjected to Chronic Heat Stress

**DOI:** 10.3390/ani16142164

**Published:** 2026-07-13

**Authors:** Shan Zhao, Xiangbin Ding, Jie Liu, Limin Wei, Jingli Yuan, Yan Zhang, Kun Ouyang, Wenbin Cao, Quanwei Liu

**Affiliations:** 1 Xia Xianzhu Academician Team Innovation Center, Key Laboratory of Tropical Animal Breeding and Disease Research, Institute of Animal Science and Veterinary Medicine, Hainan Academy of Agricultural Sciences, Haikou 571100, China; 17664411320@163.com (S.Z.); jieliu2303@163.com (J.L.); liminedu@126.com (L.W.); 13250732023@163.com (J.Y.); zy79818_0@163.com (Y.Z.); 15079542301@163.com (K.O.); caoge98322@163.com (W.C.); 2 Tianjin Key Laboratory of Agricultural Animal Breeding and Healthy Husbandry, College of Animal Science and Veterinary Medicine, Tianjin Agricultural University, Tianjin 300392, China; xiangbinding@tjau.edu.cn; 3 Sanya Research Institute, Hainan Academy of Agricultural Sciences (Hainan Experimental Animal Research Center), Sanya 572025, China

**Keywords:** Wenchang chicken, DL-methionine, heat stress, meat quality, antioxidant capacity

## Abstract

Heat stress adversely affects meat quality and antioxidant status in poultry, and nutritional interventions may help mitigate these effects. This study investigated the effects of dietary DL-methionine supplementation on meat quality, antioxidant capacity, and potential involvement of the *Keap1–Nrf2*–ARE signaling pathway in heat-stressed Wenchang chickens. A total of 480 healthy 81-day-old chickens were randomly assigned to four dietary treatments supplemented with 0.00%, 0.10%, 0.20%, or 0.40% DL-methionine and subjected to chronic heat stress conditions for 46 days. Dietary DL-methionine supplementation significantly affected meat quality and antioxidant status (*p* < 0.05). Compared with the other groups, supplementation with 0.20% DL-methionine resulted in increased cooked yield and reduced drip loss in both breast and thigh muscles. It also improved meat nutritional quality by increasing crude protein content, flavor-related amino acids, and polyunsaturated fatty acid proportions while decreasing saturated fatty acid levels. Furthermore, 0.20% DL-methionine increased the activities of superoxide dismutase, catalase, and glutathione peroxidase and reduced malondialdehyde content in breast muscle (*p* < 0.05). These changes were accompanied by altered expression of genes associated with the *Keap1–Nrf2*–ARE signaling pathway. Overall, dietary supplementation with 0.20% DL-methionine effectively improved meat quality and antioxidant capacity in Wenchang chickens under chronic heat stress conditions, whereas excessive supplementation (0.40%) showed less favorable effects.

## Simple Summary

High environmental temperatures are a major challenge for poultry production, as they can reduce meat quality and negatively affect the health of chickens. Finding effective nutritional strategies to alleviate these adverse effects is important for sustainable poultry farming. This study investigated whether dietary supplementation with different levels of methionine, an essential amino acid required for protein synthesis and metabolic regulation, could improve meat quality in Wenchang chickens raised under heat stress conditions. The results indicate that an appropriate level of methionine supplementation can reduce moisture loss in meat, increase its protein content, enhance flavor-related nutrients, and improve the balance of healthy fatty acids. Chickens receiving this optimal level of supplementation also showed improved antioxidant capacity, indicating enhanced protection against heat-induced tissue damage. However, excessive supplementation did not provide additional benefits and may adversely affect meat quality. These findings suggest that providing an appropriate level of methionine in the diet can help chickens better cope with heat stress while improving meat quality, offering a practical nutritional strategy for poultry production in high-temperature regions.

## 1. Introduction

Wenchang chicken is an indigenous poultry breed originating from Wenchang City, Hainan Province, China. It is recognized as a National Geographical Indication Product and is conserved as a vital national genetic resource due to its distinct phenotypic characteristics and superior meat quality [1]. Located at the southernmost tip of China, Hainan Island exhibits a tropical maritime monsoon climate. As a tropical island, its unique geographical and climatic conditions exert a dual impact on poultry production. On the one hand, the absence of severe cold in winter allows poultry (e.g., chickens and ducks) to allocate less energy to thermoregulation, thereby supporting maintained high growth rates and egg production rates. On the other hand, prolonged high summer temperatures (exceeding 35 °C in some areas), coupled with high humidity, readily induce heat stress in poultry, which also brings great challenges to the feeding and management of broilers [2].

Under physiological homeostasis, cells generate low levels of reactive oxygen species (ROS), such as superoxide anion radicals and hydrogen peroxide, which are efficiently neutralized by endogenous antioxidant defense systems to maintain redox equilibrium and cellular integrity. However, during heat stress, the rate of ROS production in broilers is markedly accelerated, surpassing the scavenging capacity of their antioxidant defenses and leading to a significant accumulation of these reactive molecules. This excess ROS induces pronounced oxidative stress, which subsequently inflicts oxidative damage upon critical macromolecular constituents within the cell, including proteins, lipids, carbohydrates, and DNA [3,4]. Importantly, heat stress has been demonstrated to exert systemic detrimental effects beyond muscle tissue. Recent studies have shown that heat stress disrupts intestinal redox status, alters inflammatory responses, and compromises epithelial barrier integrity in broilers, which may further exacerbate metabolic disorders and reduce nutrient utilization efficiency [5,6]. In addition, heat stress is also associated with alterations in gut microbiota composition and increased intestinal oxidative damage, highlighting its multi-organ impact on poultry health [5].

Consequently, oxidative stress under heat stress conditions has been widely recognized as a key factor contributing to impaired growth performance, dysregulated antioxidant systems, enhanced lipid and protein oxidation in muscle, reduced water-holding capacity, and deteriorated meat quality in broilers [7,8].

Methionine (Met) is not only an essential amino acid but also serves as the first limiting amino acid in corn–soybean meal-based diets for poultry. Methionine is crucial to protein synthesis, acting as the starting amino acid for translation and playing a regulatory role via its metabolite, S-adenosylmethionine (SAM). They can influence protein synthesis and cellular growth rates, impacting growth performance and meat quality of livestock and poultry [9]. Research indicates that methionine reduces oxidative stress through several mechanisms. It converts into cysteine for glutathione (GSH) synthesis, a key antioxidant [10]. SAM, a byproduct of methionine metabolism, serves as a crucial methyl donor, influencing antioxidant enzyme activity [11]. Additionally, methionine residues in proteins can directly neutralize ROS, safeguarding tissues from oxidative harm [12].

Our previous research demonstrated that dietary supplementation with an appropriate level of DL-methionine significantly improved growth performance, slaughter traits, and organ development in heat-stressed Wenchang chickens. However, its specific effects on meat quality and antioxidant function remained unclear. Therefore, the present study was designed to evaluate a range of DL-methionine doses in order to identify the optimal supplementation level for enhancing these parameters. The findings aim to inform evidence-based nutritional strategies for mitigating heat stress in Wenchang chicken production.

## 2. Materials and Methods

### 2.1. Test Material

The experimental chickens were originated from Hainan (Tanniu) Wenchang Chicken Co., Ltd. (Haikou, China). DL-methionine, with 99.00% purity, was sourced from Shandong Gusuo Biological Technology Co., Ltd., in Jining, China.

### 2.2. Experimental Animal Grouping and Diets

A total of 480 healthy 81-day-old Wenchang hens with the same genetic backgrounds and similar body weights (1141.08 ± 3.70 g) were randomly assigned to four treatment groups. Each group contained 6 replicates, with 20 birds per replicate. The birds were fed diets supplemented with 0.00%, 0.10%, 0.20%, and 0.40% DL-methionine, resulting in total methionine levels of 0.36%, 0.46%, 0.56%, and 0.76%, respectively. These supplementation levels were designed to establish a graded dose–response model, including physiological and supra-nutritional levels of DL-methionine under heat stress conditions. The experimental period lasted 46 days. The basal diet was formulated according to the nutrient requirements of yellow-feathered broilers (NY/T 3645-2020) [13]. The ingredient composition and nutrient levels of the experimental diets are presented in Table 1.

### 2.3. Temperature and Humidity During the Experimental Period

During the entire experimental period, the environmental temperature (T) and relative humidity (RH) inside the chicken house were continuously maintained under natural high-temperature conditions without artificial cooling or heating intervention. The birds were exposed to chronic heat-stress conditions throughout the trial.

Air temperature and relative humidity were monitored at three fixed time points each day (06:00, 12:00, and 18:00) using a calibrated thermo-hygrometer placed at bird level. The recorded values were used to calculate the daily Temperature–Humidity Index (THI).

The THI was calculated using the formula proposed by the National Research Council (NRC, 1971) [14]:

THI = (1.8 × T + 32) − [(0.55 − 0.0055 × RH) × (1.8 × T − 26.8)].

All environmental data were recorded throughout the experimental period to ensure accurate characterization of thermal conditions.

### 2.4. Feeding Management

The feeding trial was conducted at the Yongfa Research Base of the Institute of Animal Science and Veterinary Medicine, Hainan Academy of Agricultural Sciences. The chickens were housed in a three-tier cage. In accordance with the stocking density requirements for actual production of Wenchang chickens, each replicate group was distributed in the middle and bottom tiers of the cages, with 5 birds per cage. Each cage measured 65 cm × 44 cm × 34 cm (length × width × height), providing an effective floor area of 0.286 m^2^ per cage. Accordingly, the stocking density was approximately 17.5 birds/m^2^. A combination of supplemental artificial lighting and natural daylight was provided, along with natural ventilation. The chicken had ad libitum access to feed and water, received routine vaccinations, and were managed according to standard husbandry practices.

### 2.5. Meat Quality

To evaluate meat quality, we collected the entire breast and thigh muscles from chickens. The procedure for assessing meat quality is as follows:

(1) Samples were collected from both breast and thigh muscles, and their initial weight (W1) was accurately recorded. Each sample was placed in a cooking bag and heated in a water bath at 80 °C until the core temperature reached 75 °C, which was then maintained for 20 min. After heating, the samples were cooled to room temperature in an ice-water bath. Surface moisture was removed using filter paper, and the samples were weighed again (W2). The cooking yield (%) was calculated using the following formula:

Cooking yield (%) = (W2/W1) × 100%

(2) The pH of breast and thigh muscles was measured at 45 min (pH 45 min) and after 24 h (pH 24 h) postmortem using a FE28 Standard pH meter (METTLER TOLEDO, Zurich, Switzerland).

(3) Meat samples were taken from specified areas (e.g., chicken breast), trimmed to uniform dimensions (e.g., 2 × 2 × 2 cm cubes), and accurately weighed to obtain the initial mass (M1). The samples were placed in boil-proof bags, heated in a 75 °C constant-temperature water bath until the core temperature reached 70 °C, and then removed immediately. The cooked meat samples were cooled to room temperature, surface moisture was blotted with absorbent paper, and the final mass (M2) was immediately weighed. The result was calculated using the formula:

Cooking loss (%) = [(M1 − M2)/M1] × 100.

(4) A sample was taken from a specific location on the test muscle (e.g., chicken breast) and trimmed into a 2 cm × 2 cm × 2 cm cube. The initial mass of the meat sample (W1) was weighed using an electronic balance with an accuracy of at least 0.001 g. The meat sample was suspended in a conical flask using a fine thread, ensuring that it did not touch the flask walls. The flask opening was sealed with a self-sealing film and stored in a 4 °C refrigerator. After 24 h, the surface liquid was blotted from the meat sample with filter paper, and the sample was weighed to obtain the final weight (W2). The drip loss (%) was calculated using the formula:

Drip loss (%) = [(W1 − W2)/W1] × 100

(5) Samples were extracted from certain muscle areas and cut to standard size following the direction of the muscle fibers. After that, they were cooked and cooled using the previously described method. Next, the shear force of the sample cores was measured using a C-LM36 digital-display muscle tenderness assessment instrument (manufactured by the College of Engineering, Northeast Agricultural University, Harbin, China). We made sure that the orientation of the muscle fibers was perpendicular to the direction of the blade by placing the meat sample horizontally on the triangular slot of the shear device. In order to record the highest shear force value in Newtons (N), we turned on the device and lowered the blade at a steady speed until the meat sample was entirely severed. Ten measurements were taken for each sample, and the average was calculated to ensure the reliability of the data.

### 2.6. Muscle Histological Characteristics

Right breast and thigh muscle samples were collected immediately after slaughter from Wenchang chickens, fixed in 4% paraformaldehyde for 24 h, routinely dehydrated, paraffin-embedded, sectioned (5 μm), and stained with hematoxylin and eosin (H&E). Histological images were acquired under a light microscope at 400× magnification, and a scale bar of 100 μm was included in all images. All images were captured using identical microscope settings, and image acquisition parameters (including brightness, contrast, exposure time, and illumination intensity) were kept constant across all experimental groups to ensure comparability and minimize observational bias.

Histological images were acquired under a light microscope at 400× magnification, and a scale bar of 100 μm was included in all images. All images were captured using identical microscope settings, and image acquisition parameters (including brightness, contrast, exposure time, and illumination intensity) were kept constant across all experimental groups to ensure comparability and minimize observational bias. Magnification calibration was maintained consistently for all samples.

For each chicken, three non-overlapping sections were analyzed. Ten intact and well-oriented muscle fibers were selected from each section for muscle fiber diameter measurements, resulting in a total of 30 muscle fibers analyzed per chicken. In addition, three representative fields of view were selected from each section to determine muscle fiber number and total cross-sectional area. Fields containing damaged, folded, or obliquely oriented fibers were excluded from the analysis.

Muscle fiber diameter (μm), mean cross-sectional area (mm^2^), and muscle fiber density (cells/mm^2^) were quantified using Image-Pro Plus 6.0 software (Media Cybernetics, Rockville, MD, USA). Histological measurements were performed by an investigator blinded to the treatment groups.

### 2.7. Routine Muscle Nutrient Testing

After slaughter, the left breast and thigh muscles were uniformly collected, placed in self-sealing bags, and stored at −80 °C. Referring to GB 5009.3-2016 “National Food Safety Standard: Determination of Moisture in Food” [15], GB 5009.5-2016 “National Food Safety Standard: Determination of Protein in Food” [16], and GB 5009.6-2016 “National Food Safety Standard: Determination of Fat in Foods” [17], the moisture content, crude protein content, and intramuscular fat content in the breast and thigh muscles were determined using the drying method, the Kjeldahl method, and the ether extraction method, respectively.

### 2.8. Inosine Monophosphate Content Assay

Approximately 1 g of sample was weighed accurately to 0.0001 g, 1 mL of 50% acetonitrile (*v*/*v*, containing 0.1% hydrochloric acid) extraction solution was added, and the mixture was placed in a homogenizer for thorough homogenization. The mixture was vortex-shaken and then refrigerated at 4 °C for at least 30 min. It was centrifuged at 4 °C and 10,000 rpm for 10 min, and the supernatant was retained. Another 1 mL of extraction solution was added to the pellet, and the mixture was vortexed to perform a second extraction. Both supernatants were combined and transferred to a 10 mL brown volumetric flask for final dilution of the extract. Then, 1 mL of the solution was filtered through a 0.2 µm organic filter membrane, and the filtrate was transferred to a sample vial. The sample was stored at −80 °C for later use. Inosine monophosphate (IMP) determination in the samples was performed using high-performance liquid chromatography (HPLC; Ultimate3000-API 3200 Q TRAP, Thermo Fisher Scientific, Waltham, MA, USA), strictly following the instrument manual.

### 2.9. Detection of Amino Acid Contents in Muscle

Left breast and thigh muscles were cryopreserved at −20 °C. Following extraction, amino acid concentrations in the sample solutions were determined by peak area calculation using the external standard method, in accordance with GB 5009.124/2016 [18]. This was performed using an amino acid analyzer (post-column derivatization ion-exchange chromatograph with indophenol) for amino acid analysis.

### 2.10. Detection of Fatty Acid Contents in Muscle

Left breast and thigh muscles were cryopreserved at −20 °C. Following extraction, fatty acid content was determined using ultra-high-performance liquid chromatography (FID) as per the method specified in GB 5009.168/2016 [19]. Fatty acid content and types were calculated based on fatty acid methyl ester content and conversion factors.

### 2.11. Measurement of Muscle Antioxidant-Related Indicators

Using kits from Nanjing Jiancheng Biological Engineering Research Institute (Nanjing, China), we measured total antioxidant capacity (T-AOC; Catalog No. A015-2-1), catalase (CAT; Catalog No. A007-1-1) activity, and total superoxide dismutase (SOD; Catalog No. A001-3) activity in breast and thigh muscles. Malondialdehyde (MDA; Catalog No. BC6415) content and glutathione peroxidase (GSH-Px; Catalog No. BC1195) activity were determined using Solarbio (Beijing, China) assay kits.

### 2.12. RNA Isolation and Real-Time Quantitative PCR

We extracted total RNA from the breast muscle using the RNA Easy Fast Total RNA Extraction Kit (TIANGEN, Beijing, China), determined the concentration and purity with an Ultramicro Spectrophotometer (IMPLEN P330, Implen, Munich, Germany), reverse-transcribed and synthesized cDNA following instructions from the Reverse Transcription Kit (TIANGEN, Beijing, China), and stored it at −20 °C. Quantitative PCR amplification was conducted using MonAmp™ SYBR^®^ Green qPCR Mix (Monad Biotech, Wuhan, China), and β-actin was used as the internal reference gene to quantify the mRNA expression of *Keap1*, *Nrf2*, *SOD*, *GSH-Px* and *CAT* in breast muscle using the 2-quantitative CT method with primer sequence, synthesized by Beijing Sangon Biotechnology Co., Ltd. (Beijing, China), as shown in Table 2.

### 2.13. Western Blotting Analysis

Total protein was extracted from breast muscle tissue (*n* = 6) using RIPA lysis buffer (containing protease and phosphatase inhibitors) provided by Shanghai Sheng’er Biotechnology Co., Ltd. (Shanghai, China). Protein concentration was analyzed using the BCA Protein Assay Kit from Takara Bio, Inc. (Beijing, China), and adjusted to the same concentration as the lysis buffer. Quantified proteins were mixed proportionally with 5 × loading buffer (SB-PR037; Shanghai Sheng’er Biotechnology Co., Ltd., Shanghai, China) and boiled at 95 °C for 10 min. Protein samples were subjected to SDS-PAGE electrophoresis and transferred to polyvinylidene fluoride (PVDF) membranes (Millipore, Burlington, MA, USA) using an electrophoresis transfer apparatus. The membranes were blocked at room temperature for 20 min using Protein-Free Rapid Blocking Buffer (SB-PR079, Shanghai Sheng’er Biotechnology Co., Ltd., Shanghai, China), followed by overnight incubation at 4 °C with the primary antibody. After three washes with TBST buffer, the membranes were incubated with secondary antibody at room temperature for 1 h, followed by three additional TBST washes. Protein expression was detected using an ECL chemiluminescent kit (Affinibody Technologies Co., Ltd., Wuhan, China) with a Tanon 4600 system (Tanon, Shanghai, China). Protein bands were scanned using ImageJ software (version 1.5.4) and quantified based on optical density. Protein expression levels were normalized to β-actin as the internal loading control. Detailed information on the specific antibodies and secondary antibodies used is provided in Table 3.

### 2.14. Statistical Analysis

Statistical analyses were performed using GraphPad Prism 10.1.2 (GraphPad Software, San Diego, CA, USA). Data are presented as means ± standard deviation (SD).

Before statistical analysis, data normality was assessed using the Shapiro–Wilk test, and homogeneity of variances was evaluated using Levene’s test. One-way analysis of variance (ANOVA) was performed to evaluate the effects of different dietary DL-methionine supplementation levels. When significant differences were detected, Tukey’s multiple comparison test was used for post hoc analysis.

For meat quality, biochemical, histological, qPCR, and Western blot analyses, each pen served as the experimental unit (*n* = 6 pens per treatment group). Technical replicates (e.g., qPCR reactions) were averaged before statistical analysis and were not treated as independent observations.

Differences were considered statistically significant at *p* < 0.05.

## 3. Results

### 3.1. Temperature and Humidity

**Figure 1:**
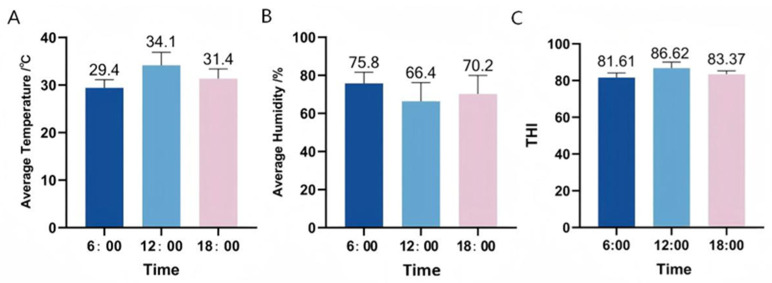
Temperature and humidity statistics during the test period. (**A**) Average temperature; (**B**) average humidity; (**C**) Temperature and Humidity Index.

Poultry heat stress levels are classified based on the Temperature–Humidity Index (THI) as follows: Comfort Period (THI < 70), Alert Period (70 < THI < 75), Danger Period (76 < THI < 81), and Emergency Period (THI > 81). Statistical results are shown in Figure 1. During the experimental period, the average temperatures in the chicken house at 06:00, 12:00, and 18:00 were 29.40 °C, 34.10 °C, and 31.40 °C, respectively. The corresponding average relative humidity values were 75.80%, 66.40%, and 70.20%, respectively. The average Temperature–Humidity Index values were 81.50, 86.62, and 83.24, respectively. Therefore, the environmental conditions experienced by the Wenchang chickens during the experimental period all indicated the presence of chronic heat stress.

### 3.2. Routine Physical Characteristics of Muscle

**Figure 2:**
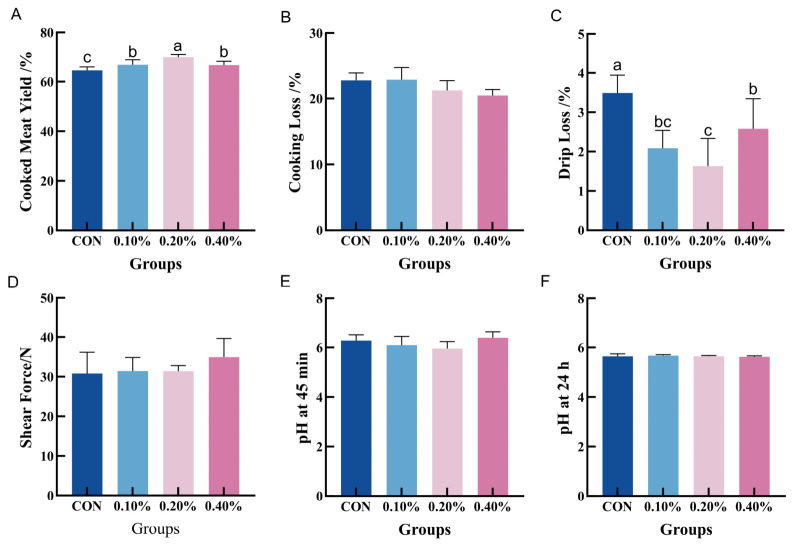
Effects of DL-methionine supplementation on conventional physical properties of Wenchang chicken breast muscle under heat stress conditions. (**A**) Cooked meat yield of the four groups; (**B**) cooking loss of the four groups; (**C**) drip loss of the four groups; (**D**) shear force of the four groups; (**E**) pH after 45 min of the four groups; (**F**) pH after 24 h of the four groups. Note: Differences marked with different lowercase letters indicate significant statistical differences (*p* < 0.05), data are presented as means ± SEM (*n* = 6).

**Figure 3:**
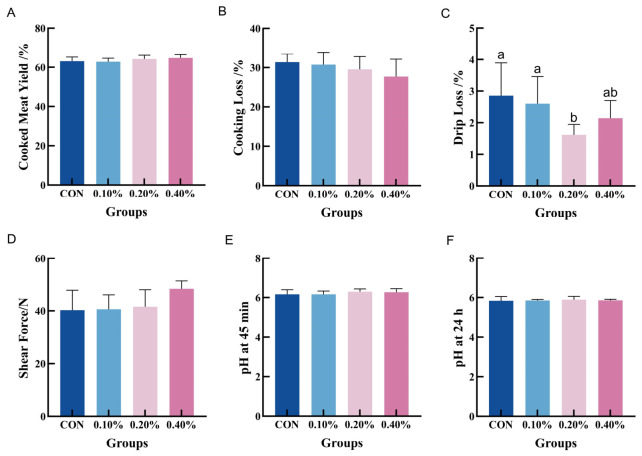
Effects of DL-methionine supplementation on conventional physical properties of Wenchang chicken thigh muscle under heat stress conditions. (**A**) Cooked meat yield of the four groups; (**B**) cooking loss of the four groups; (**C**) drip loss of the four groups; (**D**) shear force of the four groups; (**E**) pH of the four groups after 45 min; (**F**) pH of the four groups after 24 h. Note: Differences marked with different lowercase letters indicate significant statistical differences (*p* < 0.05), and data are presented as means ± SEM (*n* = 6).

As shown in Figure 2 and Figure 3, the cooked meat yield of breast muscle in the 0.20% DL-Met group was significantly higher than those in all other groups (*p* < 0.05). Yields in both the 0.10% DL-Met group and the 0.40% DL-Met group were also significantly higher than in the CON group (0.00% DL-Met group) (*p* < 0.05). Regarding drip loss, the breast muscle of the 0.20% DL-Met group exhibited significantly lower values than the CON group (0.00% DL-Met group) and the 0.40% DL-Met group (*p* < 0.05) but did not differ significantly from the 0.10% DL-Met group (*p* > 0.05). Similarly, the drip loss of thigh muscle in the 0.20% DL-Met group was significantly lower than those in the CON group (0.00% DL-Met group) and the 0.10% DL-Met group (*p* < 0.05), with no significant differences compared to the 0.40% DL-Met group (*p* > 0.05).

### 3.3. Muscle Nutrient Levels

**Figure 4:**
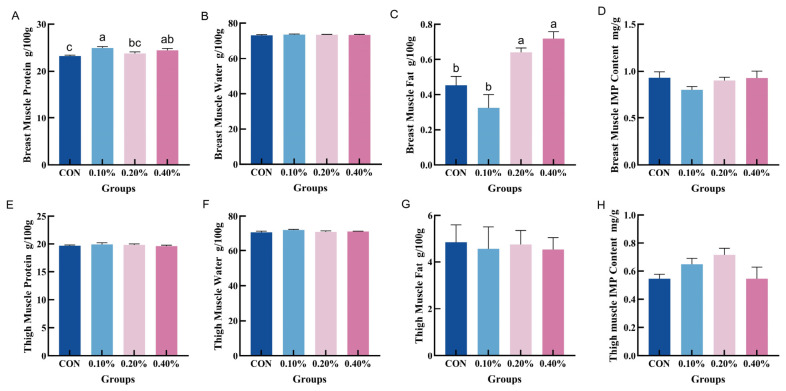
Effects of DL-methionine supplementation on muscle nutrients in Wenchang chickens under heat stress conditions. (**A**) Breast muscle protein content of the four groups; (**B**) breast muscle water content of the four groups; (**C**) breast muscle fat content of the four groups; (**D**) breast muscle adenosine monophosphate content of the four groups; (**E**) thigh muscle protein content of the four groups; (**F**) thigh muscle water content of the four groups; (**G**) thigh muscle fat content of the four groups; (**H**) thigh muscle adenosine monophosphate content of the four groups. Note: Differences marked with different lowercase letters indicate significant statistical differences (*p* < 0.05), and data are presented as means ± SEM (*n* = 6).

As shown in Figure 4, the protein content in the breast muscle of the 0.10% DL-Met group was significantly higher than those in the CON group (0.00% DL-Met group) and the 0.20% DL-Met group (*p* < 0.05) but showed no significant differences compared to the 0.40% DL-Met group (*p* > 0.05). The 0.40% DL-Met group exhibited a significantly higher protein content than the control (*p* < 0.05), while no significant differences were observed compared to the 0.20% DL-Met group. Regarding fat content, the breast muscles of the 0.20% DL-Met and 0.40% DL-Met groups contained significantly higher levels than the CON group (0.00% DL-Met group) and the 0.10% DL-Met group (*p* < 0.05). No significant differences were found between the CON group (0.00% DL-Met group) and the 0.10% DL-Met group or between the 0.20% DL-Met group and the 0.40% DL-Met group (*p* > 0.05).

### 3.4. Amino Acid Content in Muscle

**Figure 5:**
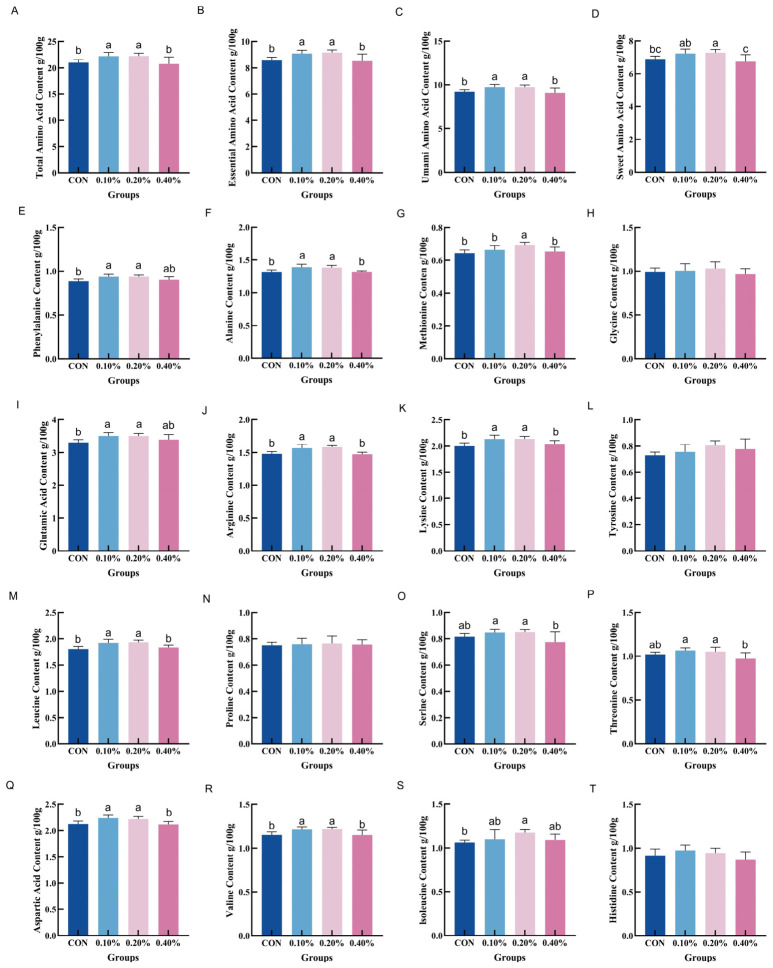
Effect of DL-methionine supplementation on amino acid content in breast muscle of Wenchang chickens under heat stress conditions. (**A**) Total amino acid content; (**B**) essential amino acid content; (**C**) umami amino acid content; (**D**) sweet amino acid content; (**E**) phenylalanine content; (**F**) alanine content; (**G**) methionine content; (**H**) glycine content; (**I**) glutamic acid content; (**J**) arginine content; (**K**) lysine content; (**L**) tyrosine content; (**M**) leucine content; (**N**) proline content; (**O**) serine content; (**P**) threonine content; (**Q**) aspartic acid content; (**R**) valine content; (**S**) isoleucine content; (**T**) histidine content. Note: Differences marked with different lowercase letters indicate significant statistical differences (*p* < 0.05), and data are presented as means ± SEM (*n* = 6).

As shown in Figure 5, in the breast muscle of the 0.10% DL-Met group and the 0.20% DL-Met group, the contents of total amino acids, essential amino acids, umami amino acids, alanine, arginine, lysine, leucine, aspartic acid, and valine were all significantly higher than those in the 0.00% DL-Met group and the 0.40% DL-Met group (*p* < 0.05). No significant differences were observed between the 0.10% DL-Met group and the 0.20% group, nor between the 0.00% DL-Met group and the 0.40% DL-Met group (*p* > 0.05). The sweet amino acid content in the 0.20% DL-Met group was significantly higher than those in the CON group (0.00% DL-Met group) and the 0.40% DL-Met group (*p* < 0.05), while no significant differences were observed compared to the 0.10% DL-Met group (*p* > 0.05). Compared with the CON group (0.00% DL-Met group), the 0.10% DL-Met group and the 0.20% DL-Met group showed significantly increased levels of phenylalanine and glutamic acid (*p* < 0.05). However, no significant differences were observed among the 0.10% DL-Met group, the 0.20% DL-Met group, and the 0.40% DL-Met group (*p* > 0.05). Regarding methionine, the 0.20% DL-Met group showed significantly higher levels than other experimental groups (*p* < 0.05), while no significant differences were observed among the other experimental groups (*p* > 0.05). Regarding serine and threonine, the 0.10% DL-Met group and the 0.20% DL-Met group showed no significant differences compared to the CON group (0.00% DL-Met group) (*p* > 0.05), but their levels were significantly higher than those of the 0.40% DL-Met group (*p* < 0.05).

**Figure 6:**
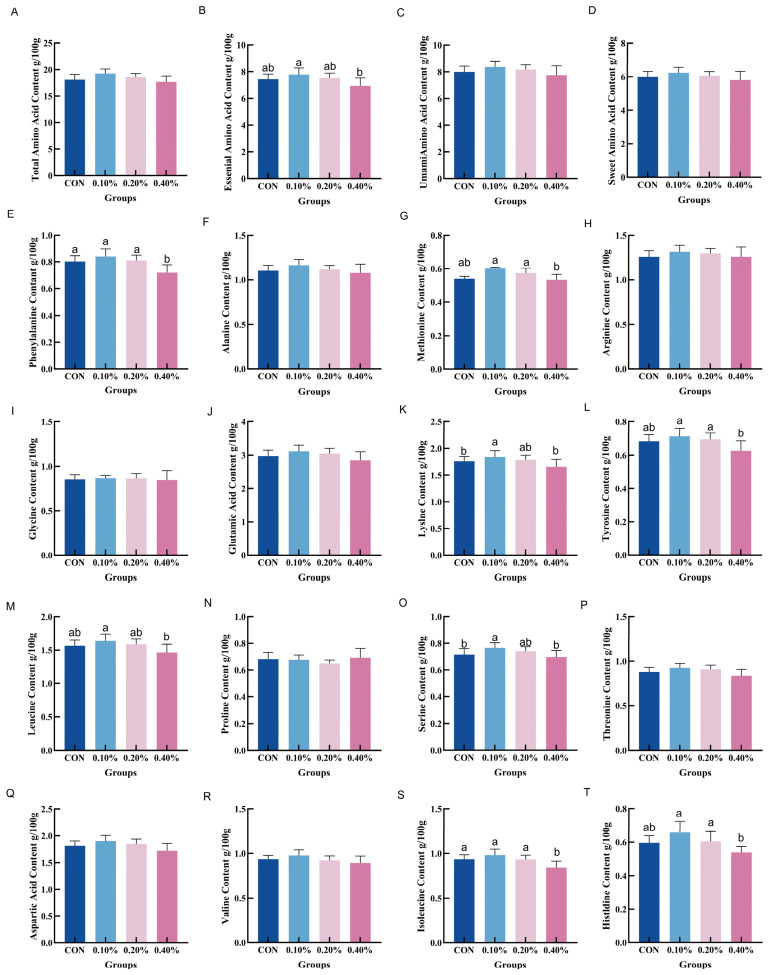
Effect of DL-methionine supplementation on amino acid content in thigh muscles of Wenchang chickens under heat stress conditions. (**A**) Total amino acid content; (**B**) essential amino acid content; (**C**) umami amino acid content; (**D**) sweet amino acid content; (**E**) phenylalanine content; (**F**) alanine content; (**G**) methionine content; (**H**) arginine content; (**I**) glycine content; (**J**) glutamic acid content; (**K**) lysine content; (**L**) tyrosine content; (**M**) leucine content; (**N**) proline content; (**O**) serine content; (**P**) threonine content; (**Q**) aspartic acid content; (**R**) valine content; (**S**) isoleucine content; (**T**) histidine content. Note: Differences marked with different lowercase letters indicate significant statistical differences (*p* < 0.05), and data are presented as means ± SEM (*n* = 6).

As shown in Figure 6, in the thigh muscle, the essential amino acid and leucine levels in the 0.10% DL-Met group were significantly higher than those in the 0.40% DL-Met group (*p* < 0.05), while no significant differences were observed compared to the CON group (0.00% DL-Met group) and the 0.20% DL-Met group (*p* > 0.05). The 0.40% DL-Met group exhibited significantly higher phenylalanine and isoleucine levels than the other experimental groups (*p* < 0.05), while no significant differences were observed among the CON group (0.00% DL-Met group), the 0.10% DL-Met group, and the 0.20% DL-Met group (*p* > 0.05). The methionine, tyrosine, and histidine levels in the 0.10% DL-Met group and the 0.20% DL-Met group were significantly higher than those in the 0.40% DL-Met group (*p* < 0.05), but no significant differences were observed compared to the CON group (0.00% DL-Met group) (*p* > 0.05). The lysine and serine levels in the 0.10% DL-Met group were significantly higher than those in the CON group (0.00% DL-Met group) and the 0.40% DL-Met group (*p* < 0.05), but no significant differences were observed compared to the 0.20% DL-Met group (*p* > 0.05).

### 3.5. The Fatty Acid Content in Muscle

**Figure 7:**
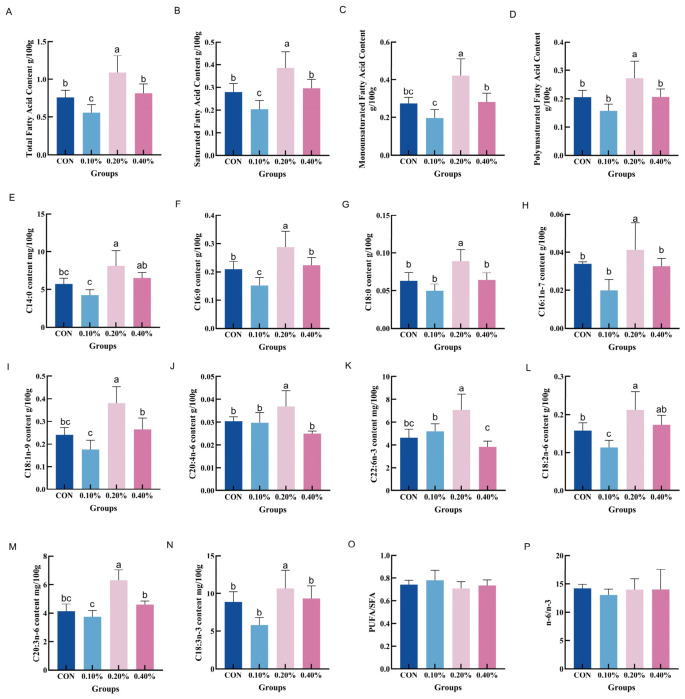
Effect of DL-methionine supplementation on fatty acid content in breast muscle of Wenchang chickens under heat stress conditions. (**A**) Total fatty acid content; (**B**) saturated fatty acid content; (**C**) monounsaturated fatty acid content; (**D**) polyunsaturated fatty acid content; (**E**) myristic acid content; (**F**) palmitic acid content; (**G**) stearic acid content; (**H**) palmitoleic acid content; (**I**) oleic acid content; (**J**) arachidonic acid content; (**K**) docosahexaenoic acid content; (**L**) linoleic acid content; (**M**) Dihomo-γ-linolenic acid content; (**N**) α-linolenic acid content; (**O**) PUFA/SFA; (**P**) n-6/n-3. Note: Differences marked with different lowercase letters indicate significant statistical differences (*p* < 0.05), and data are presented as means ± SEM (*n* = 6).

As shown in Figure 7, compared with the CON group (0.00% DL-Met group), the 0.10% DL-Met group exhibited significantly reduced levels of total fatty acids, saturated fatty acids (SFAs), and palmitic acid (C16:0) in breast muscle (*p* < 0.05); while the 0.20% DL-Met group showed significantly increased levels (*p* < 0.05). No significant differences were observed between the 0.20% DL-Met group and the 0.40% DL-Met group (*p* > 0.05). Regarding monounsaturated fatty acids (MUFAs), oleic acid (C18:1n-9), and arachidonic acid (C20:3n-6) in breast meat, the 0.20% DL-Met group showed significantly higher levels compared with the CON group (0.00% DL-Met group). The 0.10% DL-Met group and the 0.40% DL-Met group showed no significant differences compared to the CON group (0.00% DL-Met group) (*p* > 0.05). The 0.20% DL-Met group exhibited significantly higher levels of polyunsaturated fatty acids (PUFAs), stearic acid (C18:0), palmitoleic acid (C16:1n-7), arachidonic acid (C20:4n-6), and α-linolenic acid (C18:3n-3) than in the other experimental groups (*p* < 0.05), while no significant differences were observed among the other experimental groups (*p* > 0.05). Regarding myristic acid (C14:0), the 0.20% DL-Met group showed a significant increase compared with the CON group (0.00% DL-Met group) and the 0.10% DL-Met group (*p* < 0.05), while no significant differences were observed compared to the 0.40% DL-Met group (*p* > 0.05). For docosahexaenoic acid (C22:6n-3), the 0.20% DL-Met group showed a significant increase compared with the other experimental groups (*p* < 0.05). The 0.10% DL-Met group and the 0.40% DL-Met group showed no significant difference compared to the CON group (0.00% DL-Met group) (*p* > 0.05). The linoleic acid (C18:2n-6) content in the breast muscle of the 0.20% DL-Met group was significantly higher than those in the CON group (0.00% DL-Met group) and the 0.10% DL-Met group (*p* < 0.05), while no significant differences were observed compared to the 0.40% DL-Met group (*p* > 0.05). No significant differences were observed in the PUFA/SFA ratio or the n-6/n-3 ratio among the experimental groups (*p* > 0.05).

**Figure 8:**
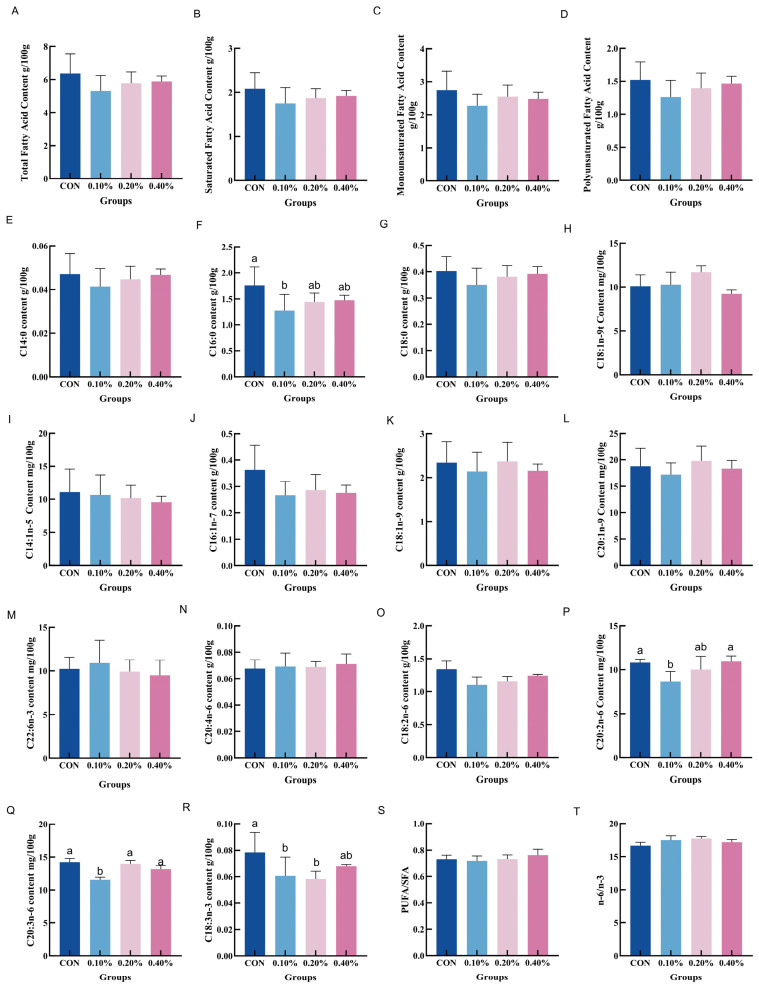
Effect of DL-methionine supplementation on thigh muscle fatty acid content in Wenchang chickens under heat stress conditions. (**A**) Total fatty acid content; (**B**) saturated fatty acid content; (**C**) monounsaturated fatty acid content; (**D**) polyunsaturated fatty acid content; (**E**) myristic acid content; (**F**) palmitic acid content; (**G**) stearic acid content; (**H**) elaidic acid content; (**I**) myristoleic acid content; (**J**) palmitoleic acid content; (**K**) oleic acid content; (**L**) gondoic acid content; (**M**) docosahexaenoic acid content; (**N**) arachidonic acid content; (**O**) linoleic acid content; (**P**) eicosadienoic acid content; (**Q**) Dihomo-γ-linolenic acid content; (**R**) α-linolenic acid content; (**S**) PUFA/SFA; (**T**) n-6/n-3. Note: Differences marked with different lowercase letters indicate significant statistical differences (*p* < 0.05), and data are presented as means ± SEM (*n* = 6).

As shown in Figure 8, the palmitic acid (C16:0) content in the thigh muscle of the 0.10% DL-Met group was significantly lower than that in the CON group (0.00% DL-Met group) (*p* < 0.05), while no significant differences were observed among the CON group (0.00% DL-Met group), the 0.20% DL-Met group, or the 0.40% DL-Met group (*p* > 0.05). Regarding docosahexaenoic acid (C20:2n-6), the thigh muscle concentration in the 0.10 DL-Met group was significantly lower than those in the CON group (0.00% DL-Met group) and the 0.40% DL-Met group (*p* < 0.05), while no significant differences were observed between the 0.10% DL-Met group and the 0.40% DL-Met group (*p* > 0.05). Regarding arachidonic acid (C20:3n-6), the 0.10% DL-Met group showed significantly higher concentrations than the other groups (*p* < 0.05), while no significant differences were observed among the other groups (*p* > 0.05). Compared with the CON group (0.00% DL-Met group), the α-linolenic acid (C18:3n-3) contents in the thigh muscles of the 0.10% DL-Met group and the 0.20% DL-Met group were significantly reduced (*p* < 0.05), while no significant differences were observed between these groups and the 0.40% DL-Met group (*p* > 0.05). No significant differences were observed in the PUFA/SFA ratio nor the n-6/n-3 ratio among the experimental groups (*p* > 0.05).

### 3.6. Muscle Morphology

**Figure 9:**
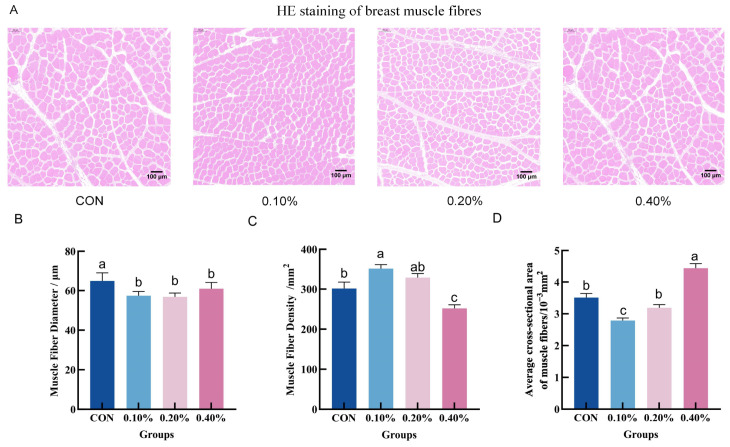
Effects of DL-methionine supplementation on histological characteristics of breast muscle tissue in Wenchang chickens under heat stress conditions. (**A**) HE-stained muscle fiber image; (**B**) muscle fiber diameter of the four groups; (**C**) muscle fiber density of the four groups; (**D**) average cross-sectional area of muscle fibers of the four groups. Note: Differences marked with different lowercase letters indicate significant statistical differences (*p* < 0.05), and data are presented as means ± SEM (*n* = 6).

**Figure 10:**
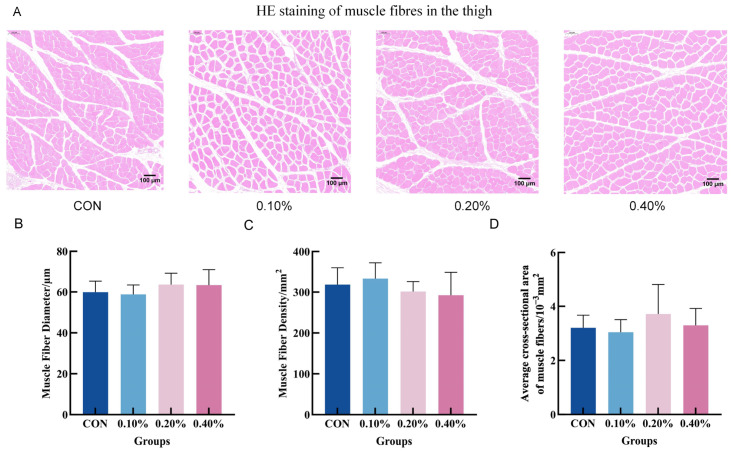
Effects of DL-methionine supplementation on histological characteristics of thigh muscle tissue in Wenchang chickens under heat stress conditions. (**A**) HE-stained muscle fiber image; (**B**) muscle fiber diameter of the four groups; (**C**) muscle fiber density of the four groups; (**D**) average cross-sectional area of muscle fibers of the four groups.

As shown in Figure 9 and Figure 10, the diameter of breast muscle fibers in the 0.10% DL-Met group, the 0.20% DL-Met group, and the 0.40% DL-Met group was significantly lower than that in the CON group (0.00% DL-Met group) (*p* < 0.05). No significant differences were observed among the 0.10% DL-Met group, the 0.20% DL-Met group, and the 0.40% DL-Met group (*p* > 0.05). The density of breast muscle fibers in the 0.10% DL-Met group was significantly higher than in the CON group (0.00% DL-Met group) and the 0.40% DL-Met group (*p* < 0.05) but showed no significant differences compared to the 0.20% DL-Met group (*p* > 0.05). Additionally, the density in the 0.40% DL-Met group was significantly lower than that in the CON group. The mean cross-sectional area of muscle fibers in the 0.10% DL-Met group was significantly lower than that in all the other groups (*p* < 0.05). The mean cross-sectional area in the 0.40% DL-Met group was significantly higher than in the CON group and the 0.20% DL-Met group (*p* < 0.05), while no significant differences were observed between the CON group and the 0.20% DL-Met group (*p* > 0.05). No significant differences were observed in muscle fiber diameter, density, or mean cross-sectional area among the groups for thigh muscles (*p* > 0.05).

### 3.7. Muscle Antioxidant Enzyme Concentration

**Figure 11:**
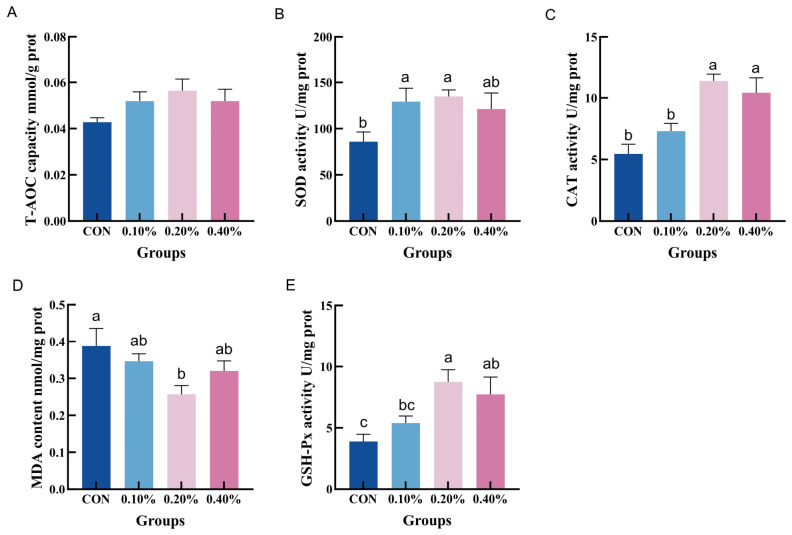
Effect of DL-methionine supplementation on antioxidant enzyme content in breast muscle of Wenchang chickens under heat stress conditions. (**A**) T-AOC levels of the four groups; (**B**) SOD content of the four groups; (**C**) CAT content of the four groups; (**D**) MDA content of the four groups; (**E**) GSH-Px content of the four groups. Note: Differences marked with different lowercase letters indicate significant statistical differences (*p* < 0.05), and data are presented as means ± SEM (*n* = 6).

As shown in Figure 11, the SOD content in the breast muscles of the 0.10% DL-Met group and the 0.20% DL-Met group was significantly higher than in the CON group (0.00% DL-Met group) (*p* < 0.05), while the difference between the 0.40% DL-Met group and the CON group did not reach a significant level (*p* > 0.05). The CAT content in the breast muscles of the 0.20% DL-Met group and the 0.40% DL-Met group was significantly higher than in the CON group and the 0.10% DL-Met group (*p* < 0.05), while the differences between the CON group and the 0.10% DL-Met group and between the 0.20% DL-Met group and the 0.40% DL-Met group were not significant (*p* > 0.05); MDA content in the breast muscle of the 0.20% DL-Met group was significantly lower than in the CON group (*p* < 0.05), while no significant differences were observed among the CON group, the 0.10% DL-Met group, and the 0.20% DL-Met group (*p* > 0.05); GSH-Px content in the breast muscle of the 0.20% DL-Met group and the 0.40% DL-Met group was significantly higher than in the CON group (*p* < 0.05), while no significant differences were found between the CON group and the 0.10% DL-Met group nor between the 0.10% DL-Met group and the 0.40% DL-Met group (*p* > 0.05).

**Figure 12:**
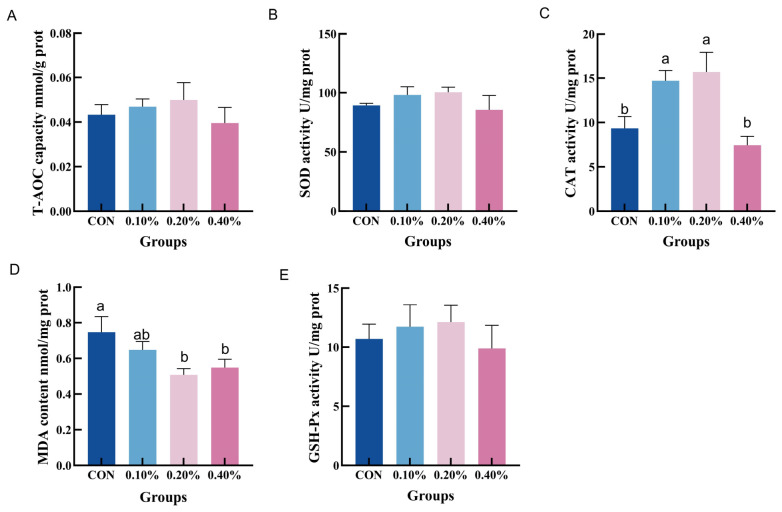
Effect of DL-methionine supplementation on antioxidant enzyme content in thigh muscles of Wenchang chickens under heat stress conditions. (**A**) T-AOC levels of the four groups; (**B**) SOD content of the four groups; (**C**) CAT content of the four groups; (**D**) MDA content of the four groups; (**E**) GSH-Px content of the four groups. Note: Differences marked with different lowercase letters indicate significant statistical differences (*p* < 0.05), and data are presented as means ± SEM (*n* = 6).

As shown in Figure 12, the CAT content in thigh muscles of the 0.10% DL-Met group and the 0.20% DL-Met group was significantly higher than in the CON group (0.00% DL-Met group) and the 0.40% DL-Met group (*p* < 0.05). However, no significant differences were observed between the CON group and the 0.40% DL-Met group nor between the 0.10% DL-Met group and the 0.20% DL-Met group (*p* > 0.05). MDA levels in thigh muscles of the 0.20% DL-Met group and the 0.40% DL-Met group were significantly lower than in the CON group (*p* < 0.05), while the difference between the CON group and the 0.10% DL-Met group did not reach statistical significance (*p* > 0.05). No significant differences were observed in MDA, GSH-Px, or T-AOC levels among thigh muscles across all groups (*p* > 0.05); data are presented as means ± SEM (*n* = 6).

### 3.8. Relative Expression Levels of Muscle Antioxidant Gene mRNAs

**Figure 13:**
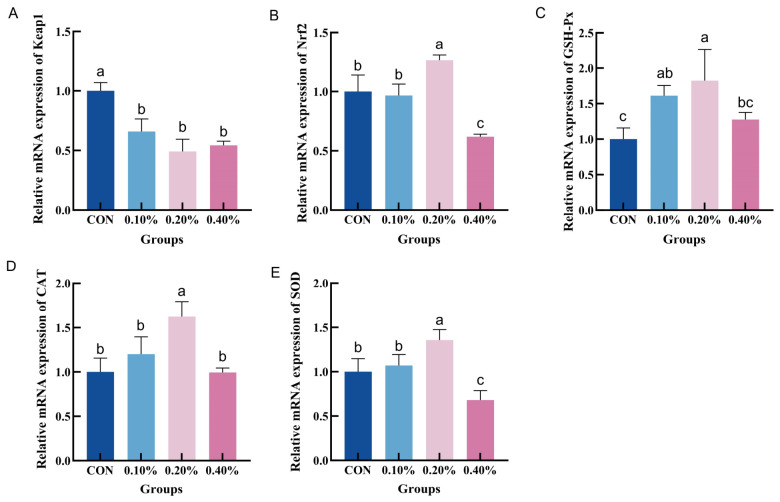
Effect of DL-methionine supplementation on relative mRNA expression levels of antioxidant-related genes in breast muscle of Wenchang chickens under heat stress conditions. (**A**) Relative expression level of *Keap1* mRNA in the four groups; (**B**) relative expression level of *Nrf2* mRNA in the four groups; (**C**) relative expression level of *GSH-Px* mRNA in the four groups; (**D**) relative expression level of *CAT* mRNA in the four groups; (**E**) relative expression level of *SOD* mRNA in the four groups. Note: Differences marked with different lowercase letters indicate significant statistical differences (*p* < 0.05), and data are presented as means ± SEM (*n* = 6).

As shown in Figure 13, in breast muscle, *Keap1* mRNA levels were significantly reduced in the 0.10%, 0.20%, and 0.40% DL-Met groups compared with the CON group (0.00% DL-Met group) (*p* < 0.05). *Nrf2* mRNA levels were significantly increased in the 0.20% DL-Met group, whereas a decrease was observed in the 0.40% DL-Met group (*p* < 0.05). *GSH-Px* mRNA levels were significantly increased in the 0.10% and 0.20% DL-Met groups compared with the CON group (0.00% DL-Met group) (*p* < 0.05). *CAT* mRNA levels were significantly increased in the 0.20% DL-Met group (*p* < 0.05), and *SOD* mRNA levels were significantly increased in the 0.20% DL-Met group, while a decrease was observed in the 0.40% DL-Met group (*p* < 0.05).

**Figure 14:**
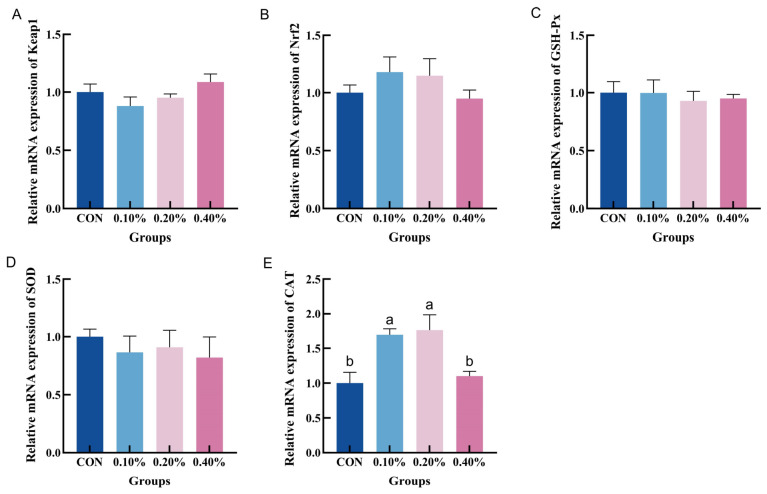
Effect of DL-methionine supplementation on relative mRNA expression levels of antioxidant-related genes in thigh muscles of Wenchang chickens under heat stress conditions. (**A**) Relative expression level of *Keap1* mRNA in the four groups; (**B**) relative expression level of *Nrf2* mRNA in the four groups; (**C**) relative expression level of *GSH-Px* mRNA in the four groups; (**D**) relative expression level of *CAT* mRNA in the four groups; (**E**) relative expression level of *SOD* mRNA in the four groups. Note: Differences marked with different lowercase letters indicate significant statistical differences (*p* < 0.05), and data are presented as means ± SEM (*n* = 6).

As shown in Figure 14, in thigh muscles, *CAT* mRNA levels were significantly increased in the 0.10% and 0.20% DL-Met groups compared with the CON group (0.00% DL-Met group) (*p* < 0.05).

### 3.9. Protein Expression Levels of Keap1 and Nrf2 in Breast Muscle

**Figure 15:**
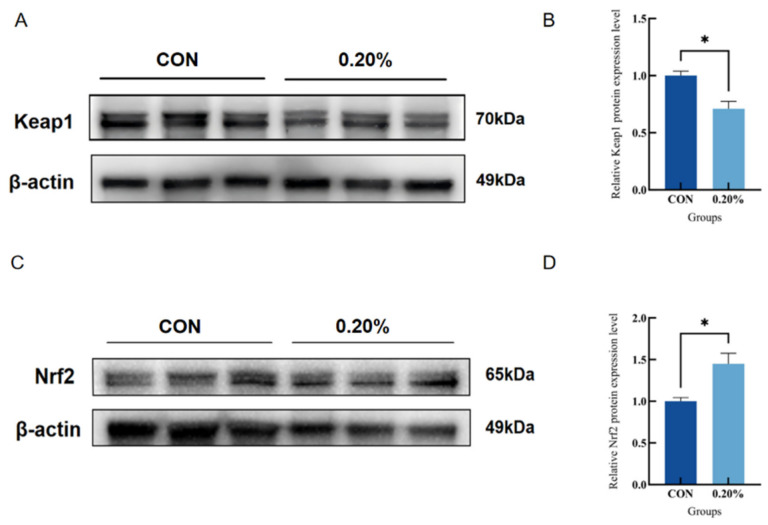
Effects of DL-methionine supplementation on Keap1 and Nrf2 protein expression levels in breast muscle of Wenchang chickens under heat stress conditions. (**A**) Representative Western blot images of Keap1 protein in breast muscle samples from Group 1 (control, 0.00%) and 0.20% (Group 3). β-Actin was used as a loading control. (**B**) Quantitative analysis of Keap1 protein expression normalized to β-actin. Data are presented as means ± SD. *p* < 0.05 vs. Group 1. (**C**) Representative Western blot images of Nrf2 protein in breast muscle samples from Group 1 (control, 0.00%) and 0.20% (Group 3). β-Actin was used as a loading control. (**D**) Quantitative analysis of Nrf2 protein expression normalized to β-actin. Data are presented as means ± SD. *p* < 0.05 vs. Group 1. Note: * Asterisks in the figure indicate significant differences compared with Group 1 (control group). *: *p* < 0.05.

As shown in Figure 15, given the aforementioned findings at the mRNA level, we further validated Keap1 and Nrf2, whose expression exhibited significant changes in breast muscle at the protein level. Western blot analysis showed that Keap1 protein expression was significantly decreased in the 0.20% DL-Met group, while Nrf2 protein expression was significantly increased compared with the CON group (0.00% DL-Met group) (*p* < 0.05).

## 4. Discussion

This study investigated how varying levels of DL-methionine supplementation in the diet affect meat quality and antioxidant capacity in Wenchang chickens under heat stress. Heat stress is a major environmental challenge in poultry production, adversely affecting growth performance, meat quality, and antioxidant status primarily through reduced feed intake and excessive oxidative stress, ultimately resulting in substantial economic losses in the poultry industry [20].

Muscle drip loss is primarily attributed to the structural disruption of myofibrillar proteins and the subsequent leakage of intracellular water and soluble proteins (e.g., myosin and sarcoplasmic proteins) during postmortem glycolysis, which weakens the integrity of the myofibrillar lattice and reduces the water-holding capacity of meat [21,22]. In addition, heat stress has been widely reported to exacerbate oxidative damage and proteolytic activity in skeletal muscle, further accelerating protein denaturation and water exudation in poultry meat [23,24]. Dietary methionine plays a central role in this process not only as a limiting essential amino acid for protein synthesis but also as a precursor for glutathione and methyl donors involved in redox regulation. Previous studies have shown that methionine supplementation or methionine derivatives can alleviate heat stress-induced cellular damage by improving antioxidant capacity and maintaining cellular homeostasis. Moreover, methionine has been reported to promote skeletal muscle growth through regulation of muscle protein metabolism-related signaling pathways, including the mTOR pathway [25,26]. Through this coordinated regulation of protein turnover and antioxidant defense, methionine helps maintain myofibrillar structural stability and reduces postmortem proteolysis, thereby improving water retention in muscle tissue [27]. Consistent with these mechanisms, the present study demonstrated that DL-methionine supplementation significantly improved meat quality in heat-stressed Wenchang chickens, as evidenced by increased cooked meat yield and reduced drip loss in both breast and thigh muscles. Notably, the most pronounced effects were observed in the 0.20% DL-methionine group, suggesting that an optimal supplementation level is required to effectively enhance muscle water-holding capacity under chronic heat stress conditions.

Oxidative stress triggers muscle protein degradation, impairs protein synthesis, and leads to muscle fiber atrophy [28]. Additionally, research has shown that methionine is a primary activator of mTOR signaling, making it a key participant in anabolic mechanisms that promote muscle protein synthesis and growth [29]. In this study, the role of DL-methionine in improving meat quality under heat stress was found to be complex and dose-dependent. Moderate supplementation with 0.20% DL-methionine most effectively promotes protein deposition through antioxidant mechanisms (potentially via the Nrf2 pathway), directly enhancing the physicochemical quality and nutritional value of muscle. Conversely, excessive supplementation may activate lipogenic pathways, altering muscle fatty acid composition and energy density, thereby potentially affecting meat flavor, tenderness, and consumer perceptions of health.

Humans must obtain essential amino acids from dietary sources, and chicken meat is recognized as a high-quality protein source that provides a balanced profile of indispensable amino acids required for human growth, development, and health [30]. In this experiment, most amino acid levels in the heat-stressed control group were low. Heat stress has been demonstrated to induce oxidative stress, which disrupts redox homeostasis and is closely associated with enhanced muscle protein degradation and suppressed protein synthesis [31], leading to a decrease in total intramuscular amino acids. Supplementing with 0.10% and 0.20% DL-methionine showed significantly higher levels of total amino acids, essential amino acids, umami amino acids, and several key amino acids (e.g., arginine, lysine, leucine, and glutamic acid) in breast muscle. This suggests that DL-methionine supplementation helps maintain muscle amino acid balance through antioxidant and protein-sparing effects. Additionally, higher levels of umami (glutamic acid and aspartic acid) and sweet (e.g., alanine and glycine) amino acids enhance the savory and sweet taste of chicken meat [32].

Heat stress-induced oxidative stress can jointly affect lipid homeostasis through two pathways: promoting lipid peroxidation reactions and regulating the expression of lipid metabolism enzyme genes [33,34]. Research has found that prolonged heat stress increases the content of saturated fatty acids in broiler chicken muscle while decreasing the content of monounsaturated and polyunsaturated fatty acids [35]. Under heat stress, unsaturated fatty acids, particularly polyunsaturated fatty acids (PUFAs), are highly susceptible to oxidative damage due to the presence of multiple double bonds, which makes them primary targets of reactive oxygen species-induced lipid peroxidation [36]. The results of this study indicate that the group supplemented with 0.10% DL-methionine effectively reduced the deposition of total fatty acids and saturated fatty acids while maintaining the stability of unsaturated fatty acids. To further evaluate the nutritional quality of muscle lipids, lipid health indices including the PUFA/SFA and n-6/n-3 ratios were calculated. The results showed that neither the PUFA/SFA ratio nor the n-6/n-3 ratio was significantly affected by DL-methionine supplementation, suggesting that although individual fatty acid components were altered, the overall balance between major fatty acid classes remained relatively stable. This demonstrates that appropriate DL-methionine supplementation can effectively alleviate heat stress-induced oxidative stress, thereby preserving the nutritional value and stability of meat quality. Additionally, the 0.20% DL-methionine supplementation group achieved the highest levels among all groups for all key fatty acid categories in breast meat, including total fatty acids, SFAs, monounsaturated fatty acids (MUFAs; e.g., oleic acid), and PUFAs (e.g., linoleic acid, α-linolenic acid, and arachidonic acid). PUFAs, which serve as precursors for chicken flavor, are essential nutrients for humans, but their molecules are unstable and highly susceptible to oxidation [37]. In this study, the 0.20% DL-methionine supplementation group showed a significant increase in polyunsaturated fatty acid content, resulting in simultaneous improvements in lipid flavor precursors and nutritional value while maintaining overall fatty acid balance.

Muscle tissue morphology serves as a key indicator for assessing muscle development [38]. Muscle fiber hypertrophy (increased diameter and cross-sectional area) represents the primary mode of muscle growth, directly correlating with an animal’s meat production performance. Meanwhile, muscle fiber density is closely associated with quality traits such as meat tenderness and juiciness [39]. This experiment found that compared with the control group, the muscle fiber diameter of the breast muscle in Wenchang chickens in the DL-methionine groups was significantly reduced. Furthermore, the breast muscle fiber density in the 0.10% DL-methionine group was significantly increased, while the mean muscle fiber cross-sectional area was significantly reduced (*p* < 0.05). It is speculated that under heat stress conditions, adding an appropriate amount of DL-methionine to the diet affects muscle development, thereby improving the tenderness of Wenchang chicken meat. However, the specific mechanism requires further experimental verification.

Heat stress disrupts redox homeostasis in broiler chickens by raising metabolic rate and stress levels, causing excess ROS production [40]. Excessive ROS can overwhelm the body’s antioxidant defenses, depleting antioxidants and disrupting the oxidative balance, leading to oxidative stress. Methionine, a sulfur-containing amino acid, is crucial to cystine synthesis, a key component of glutathione (GSH), the primary non-enzymatic antioxidant in cells that neutralizes ROS. Methionine supplementation helps reduce muscle oxidation and enhances antioxidant status in heat-stressed broilers [41]. Research has found that supplementing with methionine significantly increases GSH concentrations in muscle and liver under heat stress in broilers, enhancing their antioxidant status [42]. This study demonstrated that dietary DL-methionine supplementation significantly enhanced the antioxidant defense system in heat-stressed Wenchang chickens in a dose- and tissue-dependent manner. Specifically, in breast muscle, superoxide dismutase (SOD) activity was significantly higher in the 0.10% DL-methionine and 0.20% DL-methionine groups compared with the control group. Catalase (CAT) and glutathione peroxidase (GSH-Px) activities in breast muscle were elevated in the 0.20% DL-methionine and 0.40% DL-methionine groups. This indicates that DL-methionine activated antioxidant pathways mediated by CAT and GSH-Px. CAT decomposes hydrogen peroxide, while GSH-Px scavenges peroxides, and together they exert synergistic effects with SOD. As a glutathione (GSH) precursor, DL-methionine enhanced GSH-Px activity and potentially modulated CAT expression/activity, thereby collectively boosting antioxidant capacity. A synergistic effect of these upregulated enzymes led to a significant reduction in lipid peroxidation, indicated by lower malondialdehyde (MDA) levels in the breast muscle of the 0.20% DL-methionine group and in the thigh muscle of the 0.20% DL-methionine and 0.40% DL-methionine groups. However, at the higher 0.40% dose (Group 4), the response pattern shifted: SOD activity showed no further increase, while CAT and GSH-Px activities remained elevated. This suggests that excessive methionine intake may lead to metabolic imbalances, methyl donor overload, or amino acid antagonism; it may also induce oxidative adaptation, thereby limiting further improvements in antioxidant efficiency [43]. Tissue-specific responses were also evident, as seen in the differential activation of CAT in breast versus thigh muscle. These findings underscore that precise dosage control is critical to optimizing the antioxidant efficacy of DL-methionine supplementation in heat-stressed poultry. A major limitation is that glutathione metabolism parameters (GSH, GSSG, and GSH/GSSG ratio), which are critical downstream indicators of methionine metabolism and oxidative stress regulation, were not measured.

The *Nrf2* pathway is crucial to protecting cells from oxidative damage [44]. Heat stress significantly induces oxidative stress, which may affect *Nrf2–Keap1* interaction and lead to *Nrf2* activation. Once activated, *Nrf2* is translocated to the nucleus, where it may bind to antioxidant response elements and regulate the transcription of antioxidant genes like *SOD*, *CAT*, and *GSH-Px* [45]. These enzymes help reduce ROS and mitigate cellular oxidative damage [46]. Our findings suggest that dietary DL-methionine may alleviate heat stress-induced oxidative stress in Wenchang chickens, and modulation of the *Keap1–Nrf2*–ARE signaling pathway may be involved in this process. The significant downregulation of Keap1 and concurrent changes in *Nrf2* and antioxidant-related genes (*GSH-Px*, *CAT*, and *SOD*) in breast muscle suggest a potential involvement of this regulatory pathway. However, these events were not directly examined in the present study. It is noteworthy that the regulatory effect of DL-methionine may not follow a simple, linear dose–response relationship and may exhibit tissue specificity. In this experiment, although the 0.20% supplementation group significantly inhibited *Keap1*, *Nrf2* expression exhibited a significant downregulation, and its downstream *SOD* expression also shifted to a significant decrease. In thigh muscles, only *CAT* showed a significant response to supplementation, whereas a more pronounced change in antioxidant-related gene expression was observed in breast muscle. These observations require further investigation to clarify the underlying regulatory mechanisms.

Based on the above results, we selected breast muscle samples from the control group (0.00%, Group 1) and the 0.20% group (Group 3) for Western blot analysis of Keap1 and Nrf2. The results showed that compared with the control group (0.00%, Group 1), the Keap1 protein expression level was significantly downregulated in the 0.20% group (Group 3), while the Nrf2 protein expression level was significantly upregulated. This aligns with the transcriptional profile observed in our qPCR analysis, which showed that mRNA expression of *Nrf2* and its downstream effectors (glutathione peroxidase, catalase, and superoxide dismutase) was elevated. The consistency between transcriptional and translational data suggests that under heat stress-induced oxidative conditions, DL-methionine may be associated with modulation of the *Keap1–Nrf2* interaction, which could contribute to *Nrf2* accumulation and is accompanied by increased expression of antioxidant-related enzymes. This interpretation is consistent with previous reports suggesting that methionine-derived hydrogen sulfide may be involved in modulating *Keap1* and influencing *Nrf2* signaling, thereby contributing to antioxidant responses [47,48].

It should be noted that a limitation of the present study is the absence of a thermoneutral control group. All experimental birds were subjected to chronic heat stress conditions (THI consistently > 81), which means that the observed effects of DL-methionine supplementation were evaluated only within a heat-stressed physiological background. Therefore, the present study cannot directly determine the extent to which DL-methionine alleviates heat stress effects in comparison with birds maintained under thermoneutral conditions. Future studies incorporating thermoneutral control groups are needed to better distinguish between the restorative effects of methionine under heat stress and its role in maintaining normal physiological homeostasis. Due to the large number of variables analyzed, the possibility of type I error from multiple comparisons cannot be excluded, and the results should be interpreted with caution.

## 5. Conclusions

This study demonstrates that 0.20% dietary DL-methionine supplementation improves meat quality and antioxidant status in Wenchang chickens under chronic heat stress, potentially through modulation of antioxidant defense systems. A higher supplementation level (0.40%) did not provide further benefits under the present experimental conditions. Therefore, 0.20% DL-methionine may be considered an optimal supplementation level for Wenchang chickens exposed to chronic heat stress, within the conditions of the present study. Future studies are needed to further elucidate the underlying molecular mechanisms and long-term, tissue-specific responses to dietary methionine supplementation under heat stress.

## Figures and Tables

**Table 1:** Composition and nutrient contents of the experimental diets (dry matter basis, %).

Ingredients (%)	CON	Met 0.10%	Met 0.20%	Met 0.40%
Corn	69.45	69.45	69.45	69.45
Soybean meal	17.29	17.29	17.29	17.29
Fish meal	2.00	2.00	2.00	2.00
Wheat bran	5.00	5.00	5.00	5.00
Vegetable oil	2.91	2.91	2.91	2.91
Limestone	0.82	0.82	0.82	0.82
Dicalcium phosphate	0.97	0.97	0.97	0.97
Sodium chloride	0.30	0.30	0.30	0.30
L-lysine HCl	0.05	0.05	0.05	0.05
DL-methionine	0.11	0.21	0.31	0.51
Choline chloride	0.10	0.10	0.10	0.10
Vitamin–mineral premix ^1^	1.00	1.00	1.00	1.00
Total	100.00	100.00	100.00	100.00
Nutrient				
Metabolizable energy (MJ/kg)	12.74	12.74	12.74	12.74
Crude protein (%)	15.83	15.83	15.83	15.83
Crude fat (%)	6.11	6.11	6.11	6.11
Calcium (%)	0.83	0.83	0.83	0.83
Available phosphorus (%)	0.36	0.36	0.36	0.36
Lysine (%)	0.89	0.89	0.89	0.89
Methionine (%)	0.36	0.46	0.56	0.76
Methionine + cysteine (%)	0.63	0.73	0.83	1.03

^1^ The premix provided the following nutrients per kg of diet: Vitamin A (10,000 IU); Vitamin D (32,000 IU); Vitamin E (10 mg); Vitamin B_1_ (2 mg); Vitamin B_2_ (3 mg); Vitamin B_6_ (3.50 mg); nicotinic acid (15 mg); Cu (10 mg); Fe (80 mg); Mn (60 mg); Zn (70 mg); I (2 mg); Se (0.40 mg); Folic acid (0.50 mg); Pantothenic acid (10 mg); Biotin (0.15 mg); Cyanocobalamin (10 μg).

**Table 2:** Real-time quantitative PCR primers.

Gene	Primer Sequences (5′→3′)	Accession	bp Product Size
*Nrf2*	F: CAGGCCGTCTTGAAGCTCATCTC R:CTTGCCTCTCCTGCGTATATCTCG	NM_205117.1	179
*SOD*	F: GGTGACCTCGGCAATGTGACTG R:AATGATGCAGTGTGGTCCGGTAAG	NM_205064.2	93
*CAT*	F: CACGTATTCAGGCACTGCTGGAC R: ACGAGAAGTGGCTTGCGTGTATG	NM_001031215.2	86
*GSH-Px*	F: AAGTGCTGCTGGTGGTCAACG R: GTTGGTGGCGTTCTCCTGGTG	NM_001277853.2	155
*Keap1*	F: GATCAGCTCAACAGCACCGA R: CACGTAGATCTTGCCCTGGT	XM_042894908.1	120

**Table 3:** Antibody sources and usage.

Name	Dilute	Manufacturer	Cat
KEAP1 Rabbit pAb	1:500	ABclonal (Woburn, MA, USA)	A1820
NRF2 Rabbit pAb	1:1000	ABclonal	A11159
HRP-Conjugated Goat Anti-Rabbit lgG(H + L)	1:10,000	ABclonal	A5014
β-Actin	1:1000	Abcam (Cambridge, UK)	AB8227

## Data Availability

The data presented in this study are available upon request from the corresponding author.
